# Modeling Late-Onset Sporadic Alzheimer’s Disease Using Patient-Derived Cells: A Review

**DOI:** 10.3390/neurolint18010017

**Published:** 2026-01-14

**Authors:** Alisar Katbe, Ismaïla Diagne, Gilbert Bernier

**Affiliations:** 1 Stem Cell and Developmental Biology Laboratory, Maisonneuve-Rosemont Hospital, 5690 Boulevard Rosemont, Montreal, QC H1T 2H2, Canada; alisar.katbe@umontreal.ca (A.K.); ismaila.diagne@umontreal.ca (I.D.); 2 Department of Neurosciences, University of Montreal, Montreal, QC H3T 1J4, Canada

**Keywords:** Alzheimer’s disease, neuron, induced pluripotent stem cell (iPSC), induced neuron (iN), reprogramming, aging, epigenome

## Abstract

Late-onset sporadic Alzheimer’s disease (LOAD) is the most common form of dementia. The disease is characterized by progressive loss of memory and behavioral changes followed by neurodegeneration of all cortical areas. While the contribution of genetic and environmental factors is important, advanced aging remains the most important disease risk factor. Because LOAD does not naturally occur in most animal species, except humans, studies have traditionally relied on the use of transgenic mouse models recapitulating early-onset familial Alzheimer’s disease (EOAD). Hence, the development of more representative LOAD models through reprograming of patient-derived cells into neuronal, glial, and immune cells became a necessity to better understand the disease’s origin and pathophysiology. Herein, and focusing on neurons, we review current work in the field and compare results obtained with two different reprograming methods to generate LOAD patient’s neuronal cells: the induced pluripotent stem cell and induced neuron technologies. We also evaluate if these models can faithfully mimic cellular and molecular pathologies observed in LOAD patients’ brains.

## 1. Alzheimer’s Disease

The World Health Organization (WHO) estimates around 55 million people have dementia, out of which around 60% to 70% is attributed to late-onset sporadic Alzheimer’s Disease (LOAD) [1]. Alzheimer’s Disease (AD) is a progressive neurodegenerative disease characterized by memory and cognitive dysfunctions and behavioral changes. The disease can be generally classified into two forms, early and late onset, although the reality is more complex [2]. The early-onset form of AD (EOAD) is characterized by the onset of symptoms before the age of 65. EOAD can be sporadic or familial and is generally linked to dominant mutations in presenilin 1 (*PSEN1*), presenilin 2 (*PSEN2*) or the Amyloid Beta Precursor Protein (*APP*), although many EOAD patients do not carry defined genetic mutations [3,4,5]. LOAD is generally sporadic, with symptoms starting after the age of 65. The greatest genetic risk factor for LOAD is carrying the allele 4 of the Apolipoprotein E gene (APOE4). However, not all *APOE4* carriers develop LOAD, and *APOE2* and *APOE3* carriers can also develop LOAD [6]. LOAD is thus challenging to study because of its complexity as a multifaceted disease caused by a mix of genetic predispositions and environmental factors, and with advanced aging being the most important disease risk factor [5,7]. Importantly, LOAD does not naturally occur in most animal species studied yet, thus increasing the difficulty of studying the disease’s origin and pathological mechanisms.

### 1.1. Classical Pathological Hallmarks

Brain pathology in LOAD and EOAD is similar. It is characterized by the accumulation of extracellular amyloid plaques and of intraneuronal hyperphosphorylated Tau (p-Tau) neurofibrillary tangles in the cerebral cortex and hippocampus [8]. Plaques and tangles are generally associated with synaptic atrophy and neuronal cell death [9]. Amyloid plaques are formed by the accumulation of the 42-amino acid beta amyloid peptide (Aβ42). Aβ42 is a byproduct of APP processing toward the amyloidogenic pathway. APP, a glycoprotein essential for neuronal development, is cleaved by a β-secretase (BACE1) followed by further cleavage by the γ-secretase complex, producing the Aβ40 and Aβ42 fragments [4,10,11,12]. The Aβ42 fragment forms into insoluble fibrils at a higher rate than Aβ40, which eventually assemble to generate amyloid plaques. The amyloid hypothesis stipulates that aggregation of Aβ42 generates oxidative stress, activating multiple pathways that lead to neuronal cell death [3,13]. While neurons are the main cell type affected in AD, astrocytes also play an important role in AD pathology [14]. Among other things, they are involved in the clearance of extracellular Aβ42 through phagocytosis [15]. Tau is a microtubule-associated protein that plays an important role in axonal transport by promoting microtubule assembly through its interaction with tubulin. In healthy neurons, different post-translational modifications, including phosphorylation, are involved in Tau function. However, Tau hyperphosphorylation disrupts microtubules and promotes Tau aggregation [16,17,18,19].

### 1.2. Epigenomic Anomalies in Alzheimer’s Disease

While all our cells carry the same DNA sequence, their developmental fate, morphology, physiology, and thus identity depend on an epigenomic program that relies on DNA methylation and histone modifications to organize chromatin structure and activate or repress specific genes [20,21]. Hence, the cell identity is determined by its transcriptional program. A neuron, for example, is defined by high expression levels of neuronal-specific genes and background expression levels of non-neuronal genes (i.e., specific to muscle or skin cells). The phenomenon of loss of cell identity is when a given cell type—a neuron, for example—shows lower levels of neuronal-specific genes and higher-than-normal levels of non-neuronal genes. Interestingly, and in addition to classical AD hallmarks, numerous epigenomic anomalies have been found in LOAD brains. Cortical neurons from late-stage LOAD patients present a more relaxed chromatin characterized by loss of constitutive heterochromatin [22,23]. These compact chromatin domains correspond to gene-poor, intergenic, and repeat-containing DNA sequences of the genome that are generally methylated and labeled by the repressive histone mark H3K9me3 [24]. Loss of constitutive heterochromatin was previously linked to genomic instability, and a DNA damage response at repeat DNA sequences has been observed in LOAD brains [23]. Using next-generation sequencing, it was found that rare LOAD patients showing cognitive resilience present increased Myogenic Enhancer Factor 2C (MEF2C) gene expression [25]. MEF2C can activate neuroprotective genes such as BDNF and genes involved in axonal growth, neurotransmission and synaptogenesis such as RGS6, GABRG3 and CRHR1, suggesting that a specific epigenomic wiring may be protective against LOAD progression [26,27,28]. Using single-cell RNA sequencing (scRNA-seq) and single-nucleus ATAC sequencing (snATAC-seq) on brains from normal controls and early- and late-stage LOAD patients, it was found that LOAD is characterized by a progressive erosion of the chromatin landscape, especially that of the facultative heterochromatin normally repressed by Polycomb Repressive Complex 1 and 2 (PRC1 and PRC2) proteins [29,30]. In cortical neurons, this was linked to reduced expression of neuron-specific genes and induction of non-neuronal developmental genes, leading to loss of cell identity [18]. In this context, it is interesting to note that epigenome erosion has been proposed as an important characteristic and possible driver of cellular aging in mammals [20,31].

## 2. Materials and Methods

### 2.1. Cell Lines

Human pluripotent stem cells were approved by the “Comité de Surveillance de la Recherche sur les Cellules Souches” of the CIHR and Maisonneuve-Rosemont Hospital Ethics Committee and used by following the Canadian Institutes of Health Research (CIHR) guidelines. All methods were carried out in accordance with relevant guidelines and regulations. Controls and AD fibroblasts were obtained from clinically diagnosed individuals from Coriell Biorepository, with informed consent from all participants.

### 2.2. Differentiation of iPSCs into Cortical Neurons

The CTL2 (control) and AD2 (LOAD) iPSC lines and the neural differentiation protocol have been previous described [32,33,34]. Briefly, the Noggin agonist LDN193189 was used to reduce recombinant Noggin concentration. The iPSC lines were dissociated using Accutase (Innovative Cell Technology, San Diego, CA, USA, #AT-104) and platted on Growth Factor Reduced Matrigel (Corning, Glendale, AZ, USA, #356231) in PeproGrow hES cell media (PeproTech, Waltham, MA, USA, #BM-hESC) supplemented with ROCK inhibitor (Y-27632; 10 µM, Cayman Chemical, Ann Arbor, MI, USA, #10005583). Upon 70% of confluency, the media was changed to DDM supplemented with B27 (1X final), Noggin (10 ng/mL, Thermo Fisher Scientific, Waltham, MA, USA, #120-10C) and LDN193189 (0.5µM; Sigma-Aldrich, St-Louis, MO, USA, #SML0559) resulting in the DDM/B27/LN medium. The medium was changed every day. After 16 days of differentiation, the medium was changed to DDM/B27 and replenished every day. At day 24, neural progenitors were manually detached from the plate and platted on Growth Factor Reduced Matrigel-coated plates or chamber slides (Thermo Fisher Scientific, Waltham, MA, USA, #154534). Five days after the dissociation, half of the medium was changed for Neurobasal A media supplemented with B27 (1x final) and changed again every three days, for a total of 60 days of neural differentiation.

## 3. ATAC-Seq Data Processing Pipeline

### 3.1. Quality Control of Raw Reads

Initial quality control of paired-end FASTQ files was performed using FastQC (v0.12.1) to assess per base quality, GC content, duplication levels, and adapter contamination. Each sample (CTL and AD) was analyzed independently, and the resulting HTML reports were used to verify sequencing quality.

### 3.2. Adapter and Quality Trimming

Adapter sequences and low-quality bases were removed using Fastp (v0.24.1) with automatic adapter detection enabled (detect_adapter_for_pe). Reads shorter than 30 bp after trimming were discarded (length_required 30). The process was executed using four threads, and HTML/JSON reports were generated for each sample. The trimmed FASTQ files were stored in the trimmed/directory.

### 3.3. Alignment to the Reference Genome

Trimmed reads were aligned to the human reference genome (hg38) using Bowtie2 (v2.5.4) with the very-sensitive preset to ensure optimal alignment accuracy. Genome indices were pre-built with Bowtie2. The resulting SAM alignment files were generated for both control (CTL) and Alzheimer (AD) samples.

### 3.4. Conversion, Sorting, and Duplicate Removal

Alignment files were converted to BAM format, sorted, and indexed using SAMtools (v1.21). PCR duplicates were removed using Picard MarkDuplicates (v3.4.0) with the option REMOVE_DUPLICATES = true, and duplication metrics were written to text files (*_dup_metrics.txt). The resulting deduplicated BAM files were used for all downstream analyses.

### 3.5. Peak Calling

Accessible chromatin regions (peaks) were identified using MACS2 (v2.2.9.1) in paired-end mode (-f BAMPE), with a q-value threshold of 0.05 (-q 0.05) and the effective genome size set to human (-g hs). Peak calling was performed separately for CTL and AD samples. The resulting narrowPeak files were stored in the macs2_peaks/ directory.

### 3.6. ATAC-Seq Quality Assessment

Global ATAC-seq quality was assessed using deepTools (v3.5.6). BigWig coverage tracks were generated with bamCoverage (normalized to CPM). TSS enrichment was computed with computeMatrix in reference-point mode (±1 kb around TSS) and visualized using plotProfile-deepTools v3.5.6. Fragment size distributions were computed using bamPEFragmentSize-deepTools v3.5.6 with a maximum fragment length of 1000 bp. These quality metrics confirmed both Tn5 digestion efficiency and expected nucleosomal periodicity.

## 4. Data Analysis and Visualization

### 4.1. Global Characteristics of ATAC-Seq Peaks

Significant peaks (q < 0.05) were quantified for both CTL and AD samples using R (v4.4.3). Violin plots were generated with ggplot2 (v3.5.2) to visualize the distribution of peak surface area (width × signalValue). Chromosomal distributions of peaks were visualized as barplots to compare genome-wide accessibility between conditions.

### 4.2. Functional Annotation of Peaks

Peak annotation was performed using ChIPseeker (v1.42.0) with the annotation database TxDb.Hsapiens.UCSC.hg38.knownGene (v3.20.0) and org.Hs.eg.db (v3.20.0). Peaks were categorized according to genomic features such as promoters, introns, exons, and intergenic regions. Results were visualized using barplots and pie charts showing the relative proportion of each category for both conditions.

### 4.3. Pathway Enrichment Analysis (Reactome)

Genes associated with ATAC-seq peaks were identified using the annotatePeak function from ChIPseeker. The top 500 genes per condition (ranked by maximum signalValue) were analyzed for pathway enrichment using ReactomePA (v1.46.0). Enriched biological pathways were visualized using the dotplot and cnetplot functions from clusterProfiler (v4.14.0) and enrichplot (v1.26.1). Adjusted *p*-values were computed with the Benjamini–Hochberg correction method.

### 4.4. Software Environment

All analyses were executed within a dedicated Conda environment named atacseq_env on the Béluga high-performance computing cluster (Compute Canada). The environment included both command-line and R-based tools from the bioconda and conda-forge channels. Key dependencies and software versions are listed below:
**Software/Package****Version****Channel**FastQC0.12.1biocondaFastp0.24.1biocondaBowtie22.5.4biocondaSAMtools1.21biocondaPicard3.4.0biocondaMACS22.2.9.1biocondadeepTools3.5.6biocondabedtools2.31.1biocondaChIPseeker1.42.0biocondaTxDb.Hsapiens.UCSC.hg38.knownGene3.20.0biocondaclusterProfiler4.14.0biocondaReactomePA1.46.0biocondaorg.Hs.eg.db3.20.0biocondaGenomicRanges1.58.0biocondaggplot23.5.2conda-forgeR base4.4.3conda-forgePython3.11.12conda-forgeOpenJDK23.0.2conda-forge


### 4.5. Summary of Computational Workflow

Key steps of the workflow are listed below:
**Step****Description****Main Tools/Packages**1Raw read quality controlFastQC2Adapter and quality trimmingFastp3Alignment to hg38 genomeBowtie24BAM conversion, sorting, duplicate removalSAMtools, Picard5Peak callingMACS26ATAC-seq QC (fragment size, TSS enrichment)deepTools7Peak quantification and visualizationR, ggplot28Genomic annotation of peaksChIPseeker9Repeat element overlapRepeatMasker, GenomicRanges10Functional pathway enrichmentReactomePA, clusterProfiler


## 5. Modeling Load with Patient-Derived Cells

### 5.1. The Induced Pluripotent Stem Cell Technology

Stem cell research was revolutionized in 2006 with the development of a new method. Shinya Yamanaka laboratory succeeded at reprogramming mouse and human somatic cells into induced pluripotent stem cells (iPSCs) [35,36]. These iPSCs shared strong cellular and molecular characteristics with embryonic stem cells, including the maintenance of an extensive proliferation capacity and potential to differentiate into all cell types present in the embryo. The developed method was based on transient co-expression of the Octamer-binding Transcription Factor 4 (OCT4), SRY (sex determining region Y)-box 2 (SOX2), C-MYC, and Krüppel-like factor 4 (KLF4) genes, later referred to as Yamanaka factors [37]. Pluripotent stem cells are extremely immature based on their epigenetic state and high telomerase activity. This led to the hypothesis that reprogramming of adult fibroblasts into iPSCs erases all age-associated epigenomic marks. For example, telomere length was revealed to be generally restored to an embryonic state after reprogramming of fibroblasts into iPSCs [38]. However, extensive analyses of iPSCs produced using peripheral blood mononuclear cells from a large cohort ranging from 21 to 100 years of age revealed that some age-related marks are apparently retained from the donor’s somatic cells, including somatic mutations and methylated cytosines (CpG), the levels of which are specifically increased with age [39]. Amongst other important applications, the iPSC reprogramming discovery paved the way for modeling human development and diseases using patient-derived somatic cells.

In 2011, Israel et al. generated iPSC from two EOAD, two LOAD, and two age-matched controls using skin fibroblasts that were transfected with a Moloney murine leukemia virus (MMLV) expressing the Yamanaka factors [40]. The iPSCs were differentiated for three weeks toward a neuronal cell fate. Microtubule-associated protein 2 (MAP2) and βIII-tubulin were detected in the cells, indicating the presence of neurons. When compared to unaffected controls, ELISA assay revealed higher levels of Aβ42 in both EOAD neuronal cultures and in one of the LOAD neuronal cultures (named sAD2) (Table 1). p-Tau (Thr231) and GSK-3β levels were also higher in EOAD and sAD2 neuronal cultures when compared to normal controls, altogether revealing that iPSC-derived EOAD and LOAD neurons could recapitulate some of the classical AD pathological hallmarks. Another study performed by Kondo et al. with iPSCs reprogrammed using episomal vectors revealed that the AD neuronal pathology was partly reproductible but only in the two EOAD iPSC lines carrying mutations in *APP*, with their two LOAD iPSC lines showing no apparent pathologies upon differentiation into cortical neurons [41].

The lack of similarities between both studies using LOAD neurons suggested that a larger number of LOAD iPSC lines was required to be conclusive. In 2017, Ochalek et al. generated iPSC lines from four LOAD patients as well as from multiple EOAD (carrying PSEN1 mutations) and control patients [42]. The iPSC lines were differentiated into neurons expressing βIII-tubulin and MAP2 and the presence of glutamatergic, dopaminergic, GABAergic and cholinergic neurons was observed. Notably, it was found that secretion of Aβ40 and Aβ42 was significantly more elevated in the media of neuronal cell cultures from EOAD and LOAD when compared to controls. However, the ratio of Aβ42/Aβ40 in one LOAD culture was comparable to that of controls (Table 1). When compared to controls, GSK-3β levels were increased and Tau phosphorylation was also higher in EOAD and LOAD neuronal cultures at five different epitopes (S262, S396, S202/T205, T181 and S400/T403/S404), thus recapitulating some of the results obtained by Israel et al. The study also supports the results of Kondo et al., showing that EOAD and LOAD neurons treated with a synthetic Aβ1-42 oligomer or with H_2_O_2_ were more prone to cell death when compared to controls [41]. This study demonstrated that modeling LOAD with iPSCs differentiated into cortical neurons is feasible when using relatively mature neurons in culture.

While most studies using LOAD-derived neurons focused on classical AD pathological hallmarks, others have investigated epigenomic anomalies. Flamier et al. demonstrated a reduction in a new protein linked to the disease in their model of AD neurons differentiated from iPSCs [32]. B cell-specific Moloney murine leukemia virus integration site 1 (*BMI1*), encoding a protein part of the PRC1 [21], showed reduced gene expression and protein level in cortices and hippocampal tissues isolated from LOAD brains [32]. They differentiated four LOAD iPSC lines into MAP2 and βIII-tubulin positive neurons and found accumulation of p-Tau and Aβ42 in LOAD neurons together with reduced arborization of dendrites (Table 1). Notably, reduced BMI1 protein and gene expression were also observed in iPSC-derived LOAD neurons, thus recapitulating observations made in LOAD brains.

While multiple studies demonstrating that iPSCs differentiated into neurons could reproduce AD hallmarks in vitro, a study performed by Verheijen et al. dove into the transcriptomic comparison of LOAD neurons and LOAD-affected brain samples [43]. LOAD iPSCs were differentiated into neurons for 90 days in vitro (DIV90). RNA-seq analyses revealed 2296 Differentially Expressed Genes (DEGs) between control and LOAD neurons. Similarities with DEGs identified in LOAD brains included pathways related to Notch signaling, GABAergic neurons, synaptogenesis, mitophagy and neurodegenerative diseases.

**Figure 1:**
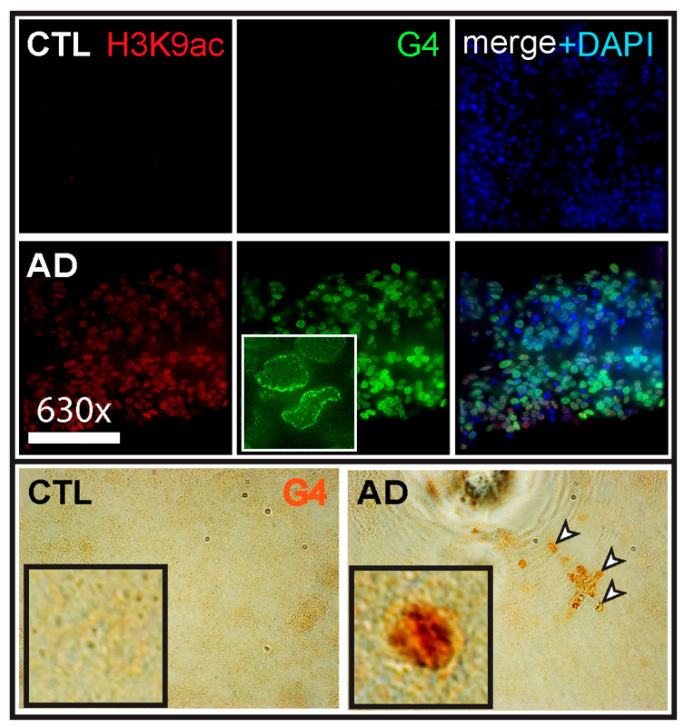
Accumulation of G4 DNA structures in cultured AD neurons and in the AD brain. Top: CTL and AD neurons produced from the differentiation of iPSCs for 60 days were labeled with an antibody for open chromatin (H3K9ac) and the 1H6 antibody against G4s (G4). Samples were analyzed by immunofluorescence. Chromatin relaxation and accumulation of G4s (inset) is observed in presumptive AD neurons. Bottom: Frozen hippocampal slices from CTL and AD cases were processed for immuno-histochemistry under non-denaturant conditions and using a G4 antibody (G4). Note the accumulation of G4s (inset) in presumptive neurons (arrowheads) of the AD case (images courtesy of A. Flamier and R. Hanna).

In another study, it was shown that relatively mature DIV60 neurons produced from LOAD iPSC lines present an excessive accumulation of non-beta DNA secondary structures called G-quadruplexes (G4s) [33]. G4s originate from single-strand DNA sequences containing tandemly spaced guanine quartets and are capable of forming a stable DNA secondary structure where guanines are linked together by Hoogsteen hydrogen bonds [47]. While G4s are enriched at gene promoters and are important to regulate transcription, they can also cause genomic instability by stalling replication forks during replication [48]. In Hanna et al., abnormal accumulation of G4 structures was found in LOAD brain sections when compared to controls [33]. Similar observations were made when analyzing cortical neurons produced from three independent LOAD iPSC lines (Figure 1). Mechanistically, it was proposed that excessive accumulation of G4s in LOAD brains and neurons originates from heterochromatin relaxation, thus promoting transcription of G4 putative sequences. ChIP-seq analysis of neurons at DIV60 further showed that most large G4 structures found in human neurons originated from the transcription of evolutionarily conserved long interspersed nuclear elements (LINEs) [49]. This revealed that abnormal accumulation of DNA secondary structures discovered in LOAD brains can be observed and thus reproduced when using iPSC-derived LOAD neurons.

In a recent study, it was revealed that epigenomic anomalies could be detected in undifferentiated LOAD iPSCs, such as reduced H3K9me3 levels (a mark of compact heterochromatin), lower *BMI1* expression, activation of stress response genes, and abnormal DNA methylation at MEF2C target genes [34]. Perturbation of heterochromatin was also found to persist at the neural progenitor and late neuronal stages. Using next-generation sequencing, LOAD iPSCs showed less efficient neural induction and a neural development phenotype characterized by mixed neuronal and glial cell identities together with reactivation of stem cell, cancer-related and cell proliferation genes in glial cells. Lower *MEF2C* expression in LOAD neurons and deregulated expression of MEF2C target genes was also observed [34]. The study raised new questions on the possible role of the epigenome at influencing patients’ vulnerability to developing LOAD by modulating the function of cognitive resilience factors such as BMI1 and MEF2C. It also recapitulated the loss of neuronal cell identity phenotype (referred as epigenomic erosion) previously described in LOAD neurons in situ.

**Figure 2:**
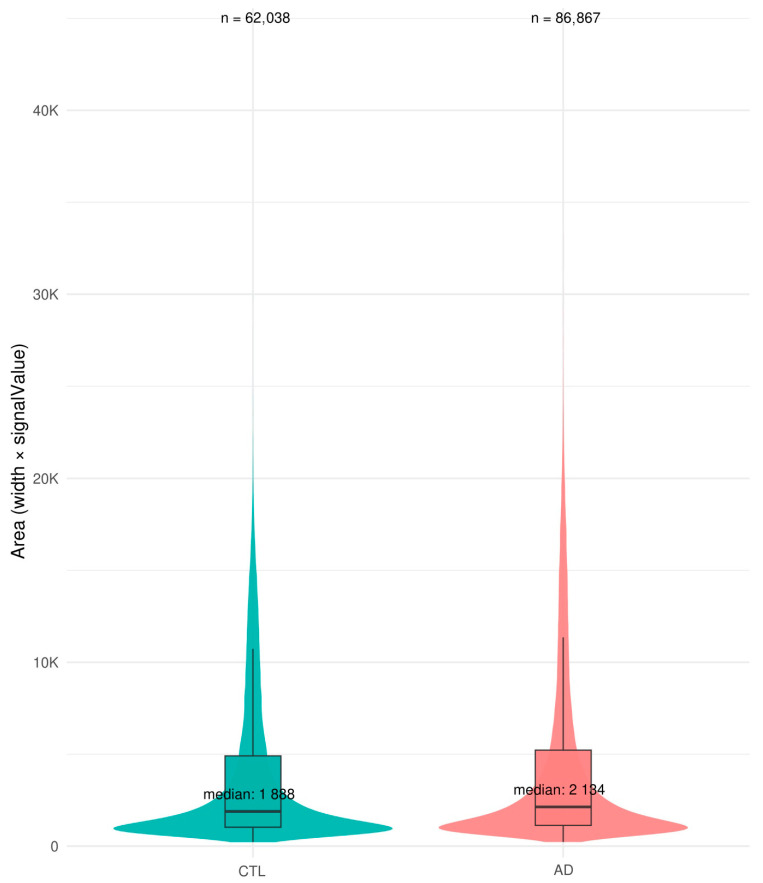
Increased chromatin relaxation in iPSC-derived LOAD neurons. Violin plot of surface area showing generally broader peaks in LOAD (AD) iPSC-derived neurons.

**Figure 3:**
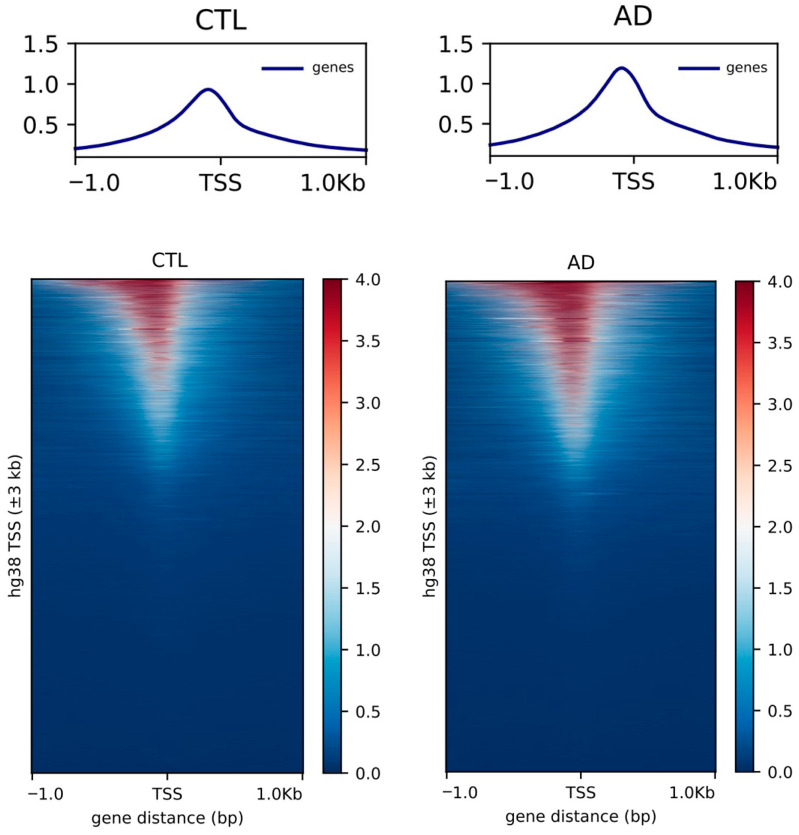
Peak distribution at TTS of genes in iPSC-derived neurons. Top: Graphical representation of the overall peaks at the Transcription Start Site (TSS) of genes for control (CTL) and LOAD (AD) neurons analyzed at DIV60. Bottom: Heatmap of peaks at Transcription Start Site (TSS) for control (CTL) and LOAD (AD) neurons.

Epigenomic anomalies described in LOAD brains implicate two phenomena that are likely, at least in part, interconnected, i.e., heterochromatin relaxation and epigenomic erosion. In a pilot study, our lab compared chromatin accessibility between DIV60 control and LOAD iPSC-derived neurons using Assay for Transposase-Chromatin Accessibility and sequencing (ATAC-seq) [50], which helps the identification of open chromatin regions. We observed a larger number of open chromatin regions in LOAD neurons (86,867 peaks) than in control neurons (62,038 peaks). Peaks also covered a larger surface and thus were generally broader (Figure 2) and were also more abundant at transcription start sites of genes in LOAD neurons (Figure 3). These results thus partly mimic the phenotype of LOAD brains which show a higher “erosion” score compared to normal control brains [29,30].

**Figure 4:**
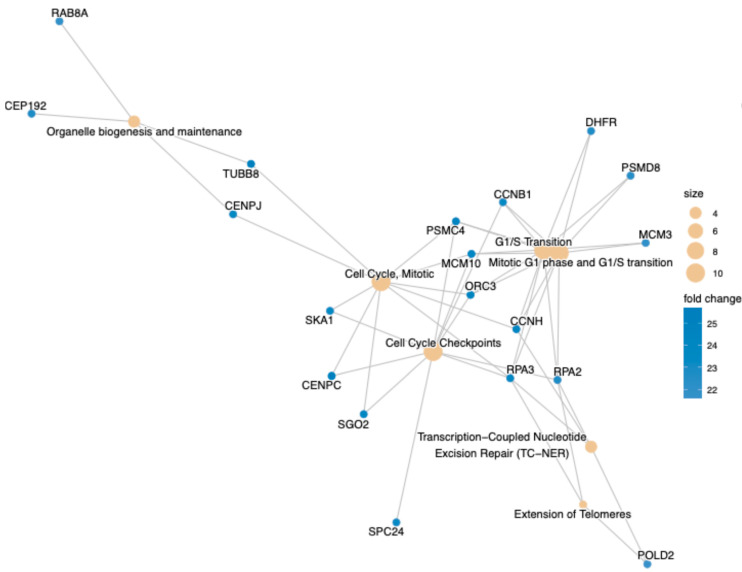
Reactome analysis of iPSC-derived control and LOAD neurons. The graph represents genes and cell biological pathways differentially activated in LOAD neurons. Note the activation of cell proliferation, cell cycle checkpoint, and DNA repair genes.

The brains’ neurons and oligodendrocytes from late-stage LOAD patients were shown to present epigenomic erosion and loss of cell identity. A “de-differentiation process” was also reported in iPSC-derived EOAD (carrying *PSEN1* mutations) neuronal cultures, although it may rather reflect a defective neural cell fate differentiation process [51]. Notably, activation of DNA repair pathways, including double-strand break repair and transcription-coupled nucleotide excision repair (TC-NER) was also found to predominate in LOAD neurons in situ when compared to neurons from cognitively normal age-match control brains [30,52]. We performed a Reactome analysis (a curated bioinformatics database of human pathways and reactions) of genes associated with differentially open chromatin in iPSC-derived control and LOAD neurons [53]. We observed enrichment for genes and pathways linked to cell proliferation, cell cycle checkpoints and TC-NER, thus partly recapitulating the chromatin accessibility phenotype observed in LOAD brains in situ (Figure 4).

Taken together, these studies demonstrated that LOAD, an age-associated disease, could be modeled using neurons derived from iPSCs, despite the fact that reprogramming into iPSCs is in principle a rejuvenation process of the cell’s epigenome [54]. A possible explanation may be related to uncharacterized genetic variants or to the presence of reprogramming-resistant LOAD-specific epigenomic anomalies that can drive the disease process.

### 5.2. The Induced Neuron Technology

A new method to study human neurons in culture and using patient-derived somatic cells was developed in 2010. Vierbuchen et al. tested different combinations of transcription factors before discovering that a combination of Achaete-Scute Homolog 1 (ASCL1), POU Class 3 Homeobox 2 (BRN2/POU3F2) and Myelin Transcription Factor 1 Like (MYT1L) genes transfected in mouse fibroblasts could efficiently convert them into induced neurons (iNs) [55]. Later, the same team showed that adding the transcription factor-encoding Neurogenic Differentiation Factor 1 (NEUROD1) gene to the other three was sufficient to generate functional and mature iNs from human fibroblasts [56]. Their study demonstrated that fibroblasts transduced with the transcription factors ASCL1 and Neurogenin-2 (NGN2) and exposed to SMAD signaling and GSK-3β inhibitors could effectively be reprogrammed into βIII-tubulin-positive neurons [57]. Using the later method, Mertens et al. analyzed gene expression of iNs obtained from young and old donors’ fibroblasts. The study revealed differences for 202 genes between iNs from young and old donors, suggesting preservation of the cellular age from the donor’s somatic cells. Next, these used primary skin fibroblasts from several controls and 13 LOAD patients to generate iNs expressing βIII-tubulin, NeuN, vGLUT and GABA [44]. Interestingly, LOAD iNs presented a similar Aβ42/Aβ40 ratio as control iNs. However, transcriptome analysis revealed up to 778 DEGs between control and LOAD iNs. That included activation of ROS, cancer-related, cell cycle and DNA damage genes, and downregulation of mature neuronal cell fate genes in LOAD iNs, altogether revealing a phenotype of de-differentiation and epigenomic erosion in LOAD iNs [44]. The study also compared iNs derived from fibroblasts to iNs derived from iPSC (iPSC-iNs) and cultured for three weeks. When looking at the transcriptomes of these neurons, iNs showed a similar profile to adult brains, whereas iPSC-iNs showed a profile similar to that of pre-natal brains [44].

Looking at two AD hallmarks, i.e., beta amyloid and p-Tau accumulation, as a focal point for LOAD modeling has been the benchmark of most studies. However, in recent years, new LOAD markers have emerged. In 2022, Herdy et al. revealed that iNs had the same senescent markers as those seen in LOAD affected brains [45]. They first studied cyclin-dependent kinase inhibitor 2A (*CDKN2A*) expression in brains from AD patients when compared to controls. *CDKN2A* encodes for a protein called p16INK4A, a tumor repressor whose role is to halt the cell cycle and proliferation in abnormal conditions, thus making it a biomarker for senescence [58]. Their results showed an increase in *CDKN2A* mRNA in the AD brains when compared to controls. Interestingly, they also showed that AD brains had three times more neurons that were NeuN/ p16INK4A-positive, which would mean a higher rate of senescence in AD compared to age-matched controls [45]. They studied the transcriptome of NeuN and βIII-tubulin positive iNs generated from LOAD and control fibroblasts. Upregulation of senescence-associated genes, including IL-6 and CDKN2A, was observed in LOAD. The increase in senescence in LOAD iNs was also demonstrated by measuring the senescence-associated beta galactosidase activity, a senescence marker [59]. This phenotype was not observed in iPSC-derived iNs [45]. Overall, Herdy et al. demonstrated that iNs could recapitulate, at least in part, the LOAD senescence phenotype observed in LOAD brains.

Although earlier studies established the potential for modeling LOAD with iNs, a detailed characterization was not achieved until 2024. The study characterized models of LOAD and EOAD using iNs to generate cortical neurons and spheroids [46]. To generate iNs, the team used an alternative method. They reprogrammed somatic cells with micro-RNA, specifically miR-9/9* and miR-124 (miR-9/9*-124), and with two transcription factors, NEUROD2 and MYT1L [46]. They generated neurons and cortical neuronal spheroids expressing MAP2 and TBR1. A significant increase in Aβ42 aggregates was detected in LOAD iNs when compared to controls, 30 days post-induction. The increase in extracellular Aβ42 was also detected in LOAD spheroids 28 days post-induction. However, high variability was observed between LOAD samples using neuronal spheroids and when compared to controls. LOAD cortical neurons at DIV30 exhibited elevated p-Tau levels when compared to controls. LOAD neurons also showed neurodegeneration, as evidenced by a significant increase in cell death when compared to controls [46]. These results were further consolidated using the TUNEL assay in LOAD spheroids. Taken together, these results demonstrated that accurate in vitro modeling of LOAD was possible using iNs and neural spheroids.

## 6. Conclusions

In this review, we summarized results obtained with two reprogramming methods used for modeling LOAD using patient-derived cells. Studies using iPSCs have demonstrated the feasibility of generating relatively mature neurons, although iPSC-derived neurons retain embryonic characteristics owing to the rejuvenation process of somatic cell reprogramming. Yet, LOAD neurons differentiated from iPSCs still exhibited AD hallmarks with increased levels of Aβ42 and p-Tau. Moreover, new molecular anomalies found to be present in LOAD brains were also reproduced using iPSC-derived LOAD neurons. Variability has also been observed between different LOAD neuronal cultures and differentiation methods, which could be partially explained by the duration of neuronal differentiation protocols used in individual studies. The iN method bypasses the iPSC stage and allows neuronal differentiation directly from somatic cells, preserving the age of the cell of origin. LOAD iNs presented several anomalies related to LOAD neurons in situ, including a senescence-associated phenotype that was not observed in iPSC-derived neurons. In conclusion, while both methods have inherent strengths and weaknesses, they allowed for the first recapitulation of the LOAD neuronal pathology using patient-derived cells, thus bypassing transgenic mouse models that better mimic EOAD.

Herein, we primarily focused on feasible and scalable 2D models to better understand LOAD. However, with advancements and development of new technologies, organogenesis is emerging as an exciting approach for modeling and investigating LOAD. Studies on cerebral brain organoids would add another level of complexity such as lamination of cortical layers [60,61]. It would also allow us to distinguish and physically localize neural progenitors, astrocytes and neurons in different cortical regions [62,63]. Furthermore, since brain organoids can be maintained for several months in culture, it could provide an interesting platform to study long-term pathological events that occur in AD, such as the apparition of amyloid plaques.

## Figures and Tables

**Table 1:** Comparative synthesis of AD pathological hallmarks between iPSC-derived neurons and iNeurons. Non-available (na) data.

Study	Model	Neuronal Markers	Extracellular Aβ	Ratio Aβ42/Aβ40	p-Tau	Oxidative Stress	Gene Expression	Epigenome	Neuronal Phenotype	Reprogramming Method
Israel et al. (2012) [40]	iPSC-neurons	MAP2, βIII-tubulin	Higher level of Aβ in EOAD and 1 LOAD	na	Higher ratio of p-Tau/Total Tau in EOAD and 1 LOAD	na	na	na	Higher volume of early and large RAB5 + endosomes	Moloney Murine leukemia virus (MMLV) vector-OCT4, SOX2, KLF4, C-MYC
Kondo et al. (2013) [41]	iPSC-neurons	SATB2 TBR1	Normal	na	na	Higher	na	na	na	Episomal vectors-OCT4, SOX2, KLF4, L-MYC + LIN28 + shP53
Ochalek et al. (2017) [42]	iPSC-neurons	MAP2, βIII-tubulin	Higher	Normal	Higher	More susceptible to H_2_O_2_	na	na	na	Sendai viral vectors-OCT4, SOX2, KLF4, C-MYC Or Episomal Vectors-OCT4, SOX2, KLF4, L-MYC + LIN28 + shP53
Flamier et al. (2018) [32]	iPSC-neurons	MAP2, βIII-tubulin	Higher	na	Higher	na	na	na	Dendritic atrophy	Episomal Vectors-OCT4, SOX2, KLF4, L-MYC + LIN28 + shP53
Hanna et al. (2021) [33]	iPSC-neurons	βIII-tubulin	na	na	na	na	RNA splicing anomalies	Chromatin relaxation & G4 structures	na	Episomal Vectors-OCT4, SOX2, KLF4, L-MYC + LIN28 + shP53
Katbe et al. (2026) [34]	iPSC-neurons	MAP2, βIII-tubulin	na	na	na	na	Downregulation of neuronal genes	Loss of hetero-chromatin	Reduced MEF2C expression	Episomal Vectors-OCT4, SOX2, KLF4, L-MYC + LIN28 + shP53
Verheijen et al. (2022) [43]	iPSC-neurons		na	Higher	na	na	Similarities with AD brains	na	na	Sendai viral vectors- OCT4, SOX2, KLF4, C-MYC
Mertens et al. (2021) [44]	iN	βIII-tubulin, NeuN	na	Normal	na	Higher	Similarities with AD brains	De-differentiation	Dendritic atrophy	Lentivirus-Ngn2:2A: Ascl1 + small molecules
Herdy et al. (2022) [45]	iN	βIII-tubulin, NeuN	na	na	na	na	Senescence and oxidative stress	Senescence genes more accessible	na	Lentivirus—Ngn2:2A: Ascl1 + small molecules
Sun et al. (2024) [46]	iN	MAP2, TBR1	Higher	na	Higher	na	Similarities with AD brains	na	Cell death/ Neurodegeneration	Lentivirus— miR-9/9*-124 + NEUROD2 and MYT1L

## Data Availability

All data can be freely obtained on demand. Accession number for the ATAC-seq at the GEO repository is: GSE314553.
